# Anti-Predator Strategies in Fish with Contrasting Shoaling Preferences Across Different Contexts

**DOI:** 10.3390/ani15233447

**Published:** 2025-11-29

**Authors:** Zixi Lu, Wuxin Li, Jiuhong Zhang, Xinbin Duan, Shijian Fu

**Affiliations:** 1 Laboratory of Evolutionary Physiology and Behavior, Chongqing Key Laboratory of Conservation and Utilization of Freshwater Fishes, Animal Biology Key Laboratory of Chongqing Education Commission of China, Chongqing Normal University, Chongqing 401331, China; 2025010513008@stu.cqnu.edu.cn (Z.L.);; 2 National Agricultural Science Observing and Experimental Station of Chongqing, Fishes Conservation and Utilization in the Upper Reaches of the Yangtze River Key Laboratory of Sichuan Province, Yangtze River Fisheries Research Institute, Chinese Academy of Fishery Science, Wuhan 430223, China; 3 Sichuan Zigong Ecological Environment Monitoring Center, Zigong 643000, China

**Keywords:** freshwater fish, anti-predator strategies, shoaling preferences, behavioral plasticity, environmental context, risk-reward trade-offs

## Abstract

In freshwater fish, group behavior is ecologically critical for daily activities such as predator avoidance. However, species with varying shoaling preferences exhibit divergent behavioral responses under different environmental conditions. This study investigated the behavioral responses of three shoaling species (*Moenkhausia costae*, *Puntius tetrazona*, and *Myxocyprinus asiaticus*) and three non-shoaling species (*Trichogaster trichopterus*, *Micropterus salmoides*, and *Cichlasoma managuense*) to simulated predation in either an open arena or a six-arm maze with shelter available. Our findings reveal that, in open water, shoaling species employ a dual strategy against predators: maintaining high group cohesion while increasing swimming speed and acceleration. This exploits the confusion effect to mitigate individual predation risk. In contrast, non-shoaling species do not engage in evasive maneuvers; instead, they adopt a cryptic strategy by minimizing activity and often freezing in place to avoid detection. In the six-arm maze, shoaling species consistently employed group coordination strategies, whereas non-shoaling species primarily relied on shelter concealment or reduced activity. Notably, shoaling species maintained high cohesion, synchronization, and activity levels across both open and complex habitats, using coordinated movement to facilitate collective escape. Together, our findings demonstrate that habitat complexity and social tendencies jointly determine how fishes trade off risk and safety. This work provides new insights into the adaptive evolution of social behavior in dynamic aquatic ecosystems.

## Simple Summary

Group-living fish often form shoals as an anti-predator strategy to reduce individual risk. This study investigates how fish species with differing shoaling preferences—shoaling and non-shoaling—adjust their anti-predator behaviors under varying conditions. In open-water environments, shoaling species form cohesive groups that help them evade predators by swimming faster and more coordinately, utilizing the “confusion effect” to confuse predators and reduce individual risk. Conversely, non-shoaling species rely on staying still to avoid detection to hide from predators. When shelter was provided in the experiments, shoaling species continued to use group coordination to escape, while non-shoaling species preferred to hide in shelters. This study highlights how environmental complexity and species’ social tendencies influence how fish balance safety and risk when facing predators. These findings provide valuable insights into fish survival strategies and the evolution of social behavior in aquatic ecosystems.

## 1. Introduction

In response to predation pressure, fish have evolved a range of anti-predator strategies that increase survival by modulating behavior to reduce risk [1,2]. A principal adaptation is shoaling. Shoaling reduces predation risk through three primary mechanisms. First, collective motion generates a “confusion effect”, reducing a predator’s ability to single out individual targets [3,4]. For example, when threatened by predators, the nearest fish initiate rapid dives and tail-fin beating, a behavior that propagates through the group. The collective motion generates surface ripples known as “fish waves”, which visually disrupt the predator, delay its attack, and thereby effectively reduce the shoal’s predation risk [5]. Second, the higher the number of shoal members, the lower the individual risk of predation (i.e., dilution effect) [6,7]. Third, information sharing and collective risk detection within groups increased prey vigilance (i.e., many eyes effect), allowing earlier predator detection and faster collective responses [8,9].

Environmental context also critically shapes anti-predator behavior. In structurally simple, open-water habitats where shelter is scarce and visibility is high, forming tight shoals and maintaining coordinated movement become primary strategies for risk mitigation [10,11,12]. In structurally complex habitats like aquatic vegetation or rocky areas, by contrast, the physical structures can limit sightlines and impede coordinated locomotion, thereby challenging the maintenance of group cohesion. Consequently, fish exhibit more diverse anti-predator strategies: they may maintain group cohesion; utilize environmental structures as refuges; or disperse to exploit the terrain for solitary concealment [13,14,15]. This trade-off between seeking refuge and maintaining collective vigilance is a key aspect of behavioral adaptation in complex environments. For instance, the presence of aquatic vegetation causes wild zebrafish (*Danio rerio*) to form less polarized and cohesive groups, as visual barriers and refuge structures diminish the need for tight social organization under predation risk [16]. Similarly, European minnows (*Phoxinus phoxinus*) form smaller, less-cohesive shoals in structurally complex habitats where refuges are abundant [12]. These studies demonstrate that environmental structure can shift anti-predator strategies along a spectrum from social to environmental reliance. This dynamic trade-off, driven by the interaction between social tendency and habitat, allows fish to flexibly optimize concealment and coordination, underscoring the remarkable adaptive diversity of piscine anti-predator behavior.

Although shoaling species’ anti-predator strategies are widely studied, it remains unclear how species with different shoaling preferences adjust their strategies under varying environmental conditions [17]. This study systematically examines these interspecific differences by comparing three shoaling species (*Moenkhausia costae*, *Puntius tetrazona* and *Myxocyprinus asiaticus*) with three non-shoaling species (*Trichogaster trichopterus*, *Micropterus salmoides* and *Cichlasoma managuense*) in contrasting experimental habitats. Shoaling species naturally exhibit cohesive group behavior [18,19,20], whereas non-shoaling species are typically solitary or form loose aggregations [21,22,23]. These distinct shoaling preferences make these species ideal models for comparative studies on anti-predator behavior. To investigate their anti-predator responses, we used an open arena and a six-arm maze to simulate homogeneous and structurally complex habitats, respectively. Based on ecological theory, we predicted that shoaling species would rely more on cohesion and coordinated escape under predation stimulation, whereas non-shoaling species would respond primarily through individual adjustments in locomotion or shelter use. Specifically, we aimed to address two questions: (1) How do shoaling and non-shoaling species deploy distinct anti-predation strategies under predation stimulation? (2) Are the anti-predation strategies of shoaling and non-shoaling species context-dependent across habitats with varying structural complexity?

## 2. Materials and Methods

### 2.1. Origin and Acclimation of Experimental Fish

In this experiment, fish were obtained from local markets and aquaculture bases (Yongchuan, Chongqing, China). After transportation, all fish were disinfected by immersion in a 1–2% NaCl solution and then transferred to separate, species-specific customized recirculating temperature-controlled tanks (2 m × 1 m × 0.5 m, water depth: 40 cm; approximately 800 L) for a 4-week acclimation period. During acclimation, ten green artificial polyethylene aquatic plants (height: 30 ± 2 cm) and gravel were placed in the tanks to provide standardized environmental enrichment. Dissolved oxygen was maintained above 7.0 mg·L^−1^ using an aeration pump, water temperature was kept at 25.0 ± 1 °C, and a 12 h light/12 h dark photoperiod was applied. Fish were fed once daily at 9:00 AM with commercial diet (Tongwei Ltd., Chengdu, Sichuan, China). Fish were fed to apparent satiation, and uneaten feed and feces were removed 30 min after feeding by siphoning, and approximately 20% of the tank water was replaced with dechlorinated tap water that had been aerated for 48 h.

In this study, all animal procedures strictly complied with: Ethical standards and animal care guidelines of Chongqing Key Laboratory of Conservation and Utilization of Freshwater Fishes, China (Permit No. Fu2020112302), National standards for laboratory animal facilities (GB/T14925-2001) [24], and Chongqing Municipal Regulations on Experimental Animal Administration.

### 2.2. Experimental Protocol and Determination

After acclimation, fish of similar size and good health were selected for the experiments. All six fish species were fasted for 24 h prior to the experiments to prevent interference from digestive activities. All experiments were recorded between 08:00 AM and 17:00 PM to minimize the potential influence of circadian rhythms. Our study comprised two experiments. Experiment 1 examined shoaling behavior under predation risk in an open-water environment to compare interspecific differences. Experiment 2 investigated the effects of predation risk on spatial distribution and group dynamics in a six-arm maze to evaluate context-dependent strategies.

#### 2.2.1. Experimental Device of Group Behavior in Open-Water Arena

**Figure 1:**
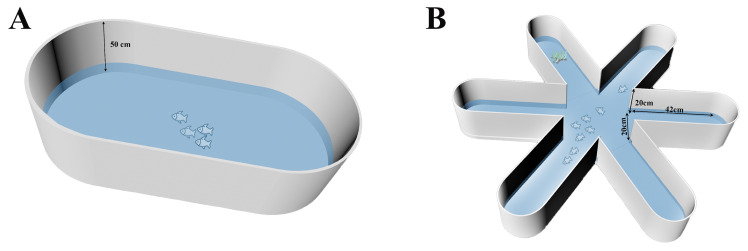
Experimental arenas for anti-predator behavioral assays in fish. (**A**) Open-water arena. (**B**) Six-arm radial maze.

The open-water shoaling experiment utilized an oval white plastic tank (2 m × 1 m × 0.5 m; Figure 1A), a design chosen to minimize corner aggregation and reduce external disturbances. A high-definition wide-angle camera (Canon BMPCC 6K, camera shot: Canon EF16-35mm f/4L IS USM, Canon U.S.A., Inc., Melville, NY, USA) was mounted directly above the tank and connected to a computer to record fish behavior. The tank was surrounded by white opaque curtains to minimize disturbances from external activity, and the laboratory walls and floor were covered with sound-absorbing materials to eliminate noise interference.

A total of 480 fish (80 per species) were used, with the following mean body measurements: *Moenkhausia costae* (body weight: 1.00 ± 0.04, body length: 3.75 ± 0.04); *Puntius tetrazona* (body weight: 1.22 ± 0.04, body length: 3.37 ± 0.03); *Myxocyprinus asiaticus* (body weight: 2.19 ± 0.11, body length: 4.67 ± 0.07); *Trichogaster trichopterus* (body weight: 2.96 ± 0.13, body length: 5.11 ± 0.07); *Micropterus salmoides* (body weight: 0.93 ± 0.02, body length: 3.94 ± 0.03); and *Cichlasoma managuense* (weight: 1.62 ± 0.15, length: 3.72 ± 0.12). Each species was divided into 20 groups (*n* = 20), with 4 fish per group. No fish were reused in the experiments. Briefly, each group was gently transferred to the tank using a small opaque glass beaker to acclimate for 10 min. After the acclimation period, the experiment commenced with a 10 min video recording session. A predator attack was simulated during the 5th minute of filming by manipulating model of a little egret (*Egretta garzetta*, 28 cm in length) attached to a 40 cm long metal rod was hovered above the water surface and then plunged downward to mimic a diving predator. After the trials, all videos were converted to “.avi” format at 15 frames·s^−1^ using Format Factory software (V5.21.0.0). Individual trajectories and group-level dynamics were extracted frame by frame using idtracker.ai (v5.2.6) [25,26] implemented in Python 3.11.7.

In this experiment, we focused on several parameters to characterize fish swimming behavior: instantaneous speed, swimming acceleration, synchronization of speed, inter-individual distance, polarity, and percent time spent moving. Swimming speed, swimming acceleration, and percent time spent moving together represent the swimming capacity of the fish. Swimming acceleration reflects the group’s ability to accelerate, which is indicative of high-energy movement patterns in the fish. percent time spent moving, on the other hand, reflects the proportion of time spent in active movement, serving as an indicator of the group’s spontaneous swimming activity. The cohesion of the shoal is further assessed through synchronization of speed, polarity, and inter-individual distance. Specifically, synchronization of speed and polarity both reflect the level of coordination within the shoal, while inter-individual distance represents the average distance (cm) between all individuals in the shoal and evaluates group cohesion. The smaller the value, the stronger the group cohesion [27]. The relevant parameters were calculated as described in Table 1.

#### 2.2.2. Experimental Device of Group Behavior in Six-Arm Maze

Shoaling behavior was assessed using a radial six-arm maze (80 cm length × 20 cm width × 20 cm height, with a water depth of 10 cm, Figure 1B). The six-arm radial maze consisted of a central hexagon (each side: length 20 cm × 20 cm height) and six radial arms (each arm: 42 cm length × 20 cm width × 20 cm height), a design that prevented fish from aggregating in corners. The maze was surrounded by white opaque curtains to minimize disturbances from surrounding activity. All trials were recorded from directly overhead using a high-definition camera (Logitech C920, Logitech, Shanghai, China; 30 fps) connected to a computer. For each species, a total of 160 fish were used, divided into 20 groups (*n* = 8 individuals per group). Briefly, each group was gently transferred to white opaque cylindrical adapters (24 cm in diameter) located in the central area of the maze using a small opaque glass beaker. After a 10 min acclimation period, the adapters were removed to initiate the trial. Spontaneous swimming was recorded for 15 min, during which a simulated predator attack (method detailed in Section 2.2.1) was applied at the 15th minute. Recording continued for a final 5 min post-stimulus, yielding a total recording duration of 20 min. After the experiment, all videos were converted to an appropriate format. The number of fish in the central area and in each arm of the maze was analyzed frame by frame using a maze counting system developed in MATLAB (vR2020b).

In this experiment, two types of parameters were extracted: density and group dynamic parameters. Density was defined as the average number of fish per frame in each region of the maze (shelter arm, normal arms and central area). Group dynamic parameters included the cohesion index (*I_C_*), shuttling frequency and the percentage of time spent in a group. Fish were considered to form a group when the number of individuals in an arm was greater than or equal to (*n* + 1)/2 (in this experiment, ≥5 fish). Central density refers to the distribution density of fish in the central area. Shuttling frequency (times·min^−1^) was the number of times per minute a fish group moved from one arm to another. *I_c_* indicated the ability of animals to form cohesive groups in the radial maze. *I_c_* varied between 0 and 1, increasing as the number of occupied zones decreased and the groups became larger. The formula for calculating *I_c_* is given below [31].

$$I_{C}=(D_{c}-D_{min})/(N-D_{min})$$

where *D_c_* is the Euclidean distance between the number of individuals (*f_i_*) in each area of the maze and *D_min_* is the minimum *D_c_* value, i.e., the value of the fish group in its most dispersed state. *N* is the total number of fish. The calculation formula for *D_c_* is shown below:

$$D_{c}=\sqrt{\sum_{i=1}^{N}{\left(f_{i}\right)}^{2}}$$

where *f_i_* is the number of fish per area in the maze. *N* is the total number of fish.

### 2.3. Statistical Analysis

All experimental data were initially processed and calculated using Excel 2019, followed by analysis with SPSS 26.0. Data are presented as the mean ± SE, with the significance level set at *p* < 0.05 for all tests. Normality and homogeneity of variance were assessed using the Kolmogorov–Smirnov test. Subsequently, linear mixed models (LMMs) were employed to examine the effects of shoaling preference and simulated predation stimulation on behavioral parameters, including instantaneous speed, instantaneous acceleration, percent of time spent moving, inter-individual distance, synchronization of speed, polarity, center density, shelter density, *I_C_*, and shuttling frequency during both open-water and six-arm maze trials. The models included shoaling preference and predation stimulation as fixed effects, behavioral parameters as dependent variables, and species as a random factor. When LMMs revealed significant differences among or within categories, one-way ANOVA was conducted to investigate parameter differences between spontaneous and simulated predation conditions, followed by Bonferroni correction.

## 3. Results

### 3.1. Behavioral Responses in Open-Water Arena

**Figure 2:**
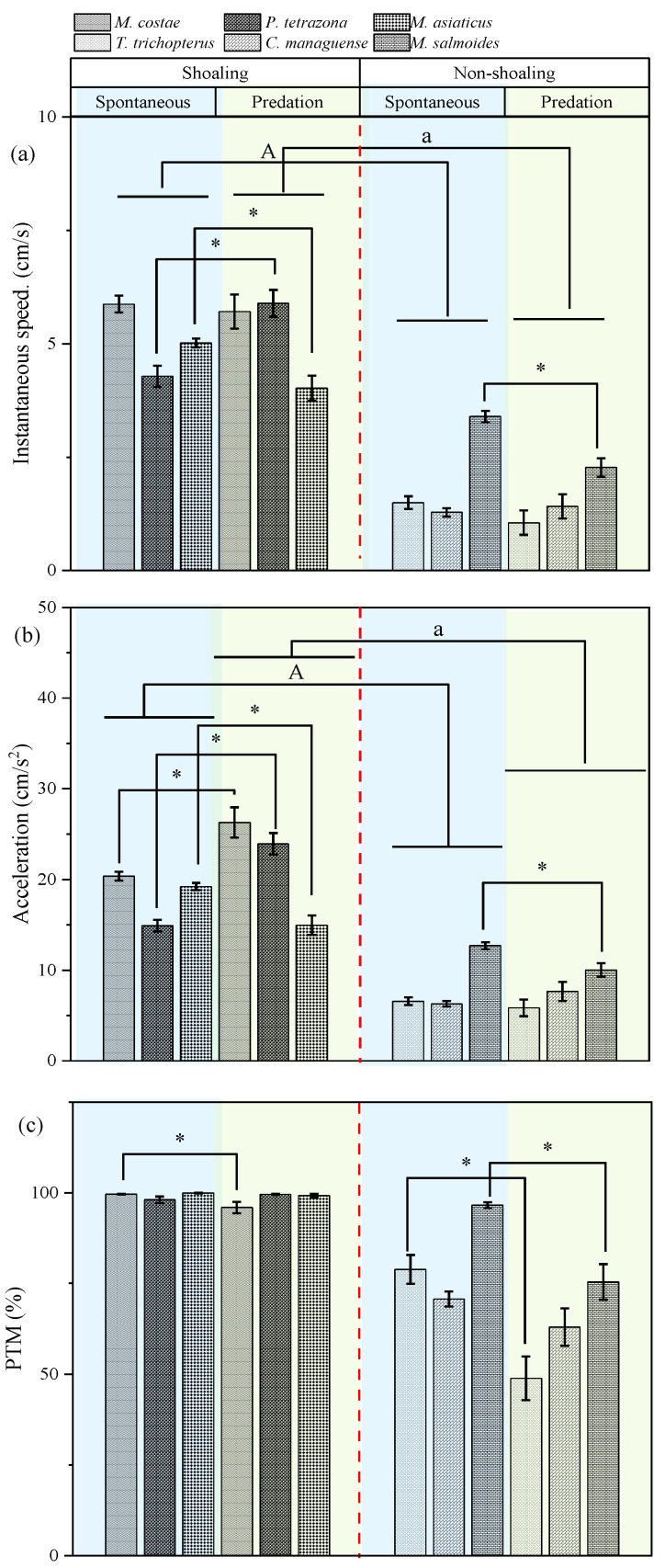

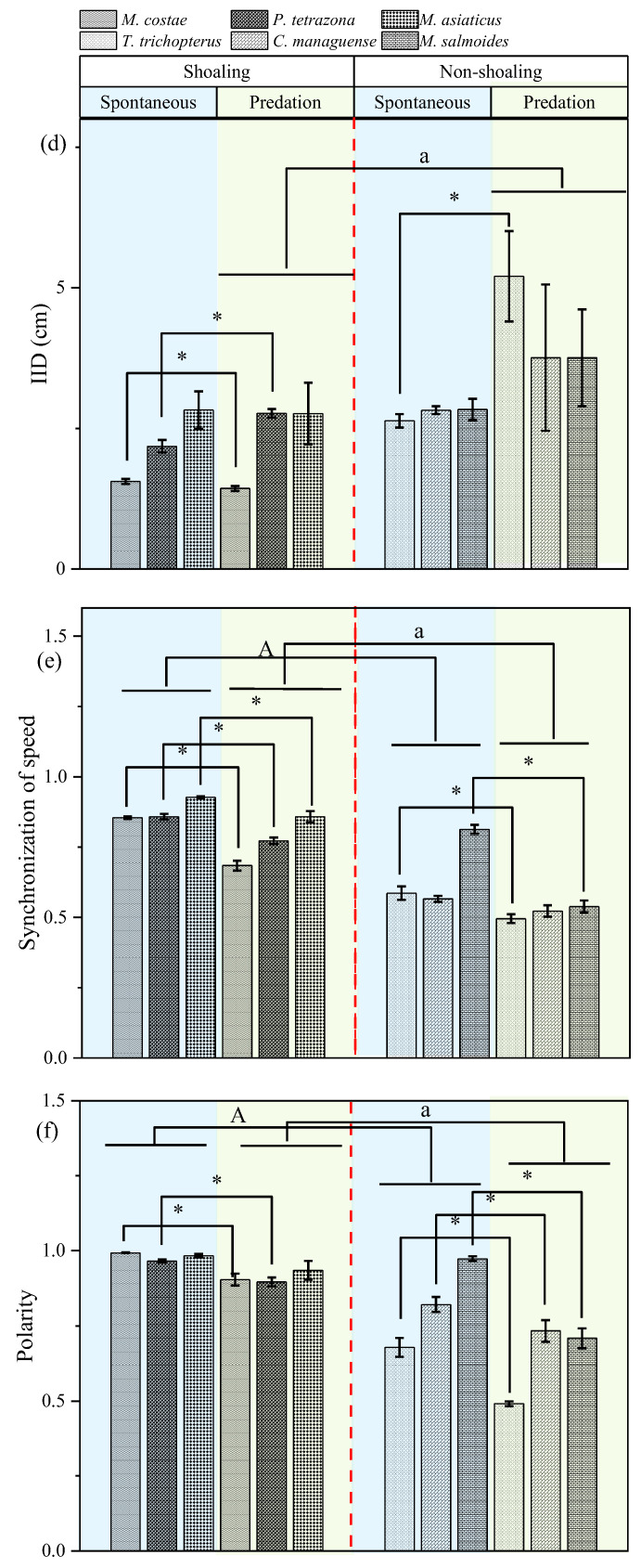
Changes in shoaling behavior parameters of fish with different shoaling preferences before and after simulated predation stimulation in the open-water arena (mean ± S.E.). Notes: The capital letters (A) indicate significant differences between shoaling-preference groups under spontaneous conditions (*p* < 0.05); The lowercase letter (a) indicate significant differences between shoaling-preference groups under predation stimulation (*p* < 0.05). The star-shaped symbol (*) indicate significant differences within species before and after predation stimulation (*p* < 0.05).

**Instantaneous speed:** Shoaling preference (*p* < 0.006) had a significant effect on instantaneous speed, and this effect varied with predation stimulation (interaction: *p* = 0.033). Shoaling species maintained significantly higher speeds than non-shoaling species both before and after predation stimulation (*p* < 0.05). Predation stimulation induced a significantly higher speed in one shoaling species (*P. tetrazona*), but significantly lower speeds in one shoaling species (*M. asiaticus*) and one non-shoaling species (*M. salmoides*) (*p* < 0.05) (Table 2, Figure 2a).

**Instantaneous Acceleration:** Both shoaling preference (*p* = 0.008) and predation stimulation (*p* = 0.014) had significant effects on acceleration, and their interaction was also significant (*p* < 0.001). Shoaling species exhibited significantly higher acceleration than non-shoaling species both before and after predation stimulation (*p* < 0.05). Predation stimulation significantly increased acceleration in two shoaling species (*M. costae* and *P. tetrazona*), but decreased it in one shoaling species (*M. asiaticus*) and one non-shoaling species (Table 2, Figure 2b).

**Percent time spent moving (PTM):** Both shoaling preference (*p* = 0.019) and predation stimulation (*p* < 0.001) had significant effects on PTM, and their interaction was also significant (*p* < 0.001). Predation stimulation significantly reduced PTM in one shoaling species (*M. asiaticus*) and two non-shoaling species (*T. trichopterus* and *M. salmoides*) (*p* < 0.05) (Table 2, Figure 2c).

**Inter-individual distance (IID):** Both shoaling preference (*p* = 0.048) and predation stimulation (*p* < 0.001) had significant effects on IID, and their interaction was also significant (*p* = 0.039). After predation stimulation, non-shoaling species showed significantly greater IID than shoaling species (*p* < 0.05). Predation stimulation significantly increased IID in one shoaling species (*P. tetrazona*) and one non-shoaling species (*T. trichopterus*), but significantly decreased it in one shoaling species (*M. costae*) (*p* < 0.05) (Table 2, Figure 2d).

**Synchronization of speed:** Both shoaling preference (*p* = 0.014) and predation stimulation (*p* < 0.001) had significant effects on synchronization of speed. Shoaling species maintained significantly higher synchronization than non-shoaling species both before and after predation stimulation (*p* < 0.05). Predation stimulation significantly reduced synchronization in all shoaling species (*M. costae*, *P. tetrazona* and *M. asiaticus*) and in two non-shoaling species (*T. trichopterus* and *M. salmoides*) (*p* < 0.05) (Table 2, Figure 2e).

**Polarity:** Predation stimulation had a significant effect on polarity (*p* < 0.001), and this effect varied with shoaling preference (*p* < 0.01). Shoaling species maintained significantly higher polarity than non-shoaling species both before and after predation stimulation (*p* < 0.05). Predation stimulation significantly reduced polarity in two shoaling species (*M. costae* and *P. tetrazona*) and in all non-shoaling species (*T. trichopterus*, *C. managuense* and *M. salmoides*) (*p* < 0.05) (Table 2, Figure 2f).

### 3.2. Behavioral Responses in Six-Arm Maze

**Figure 3:**
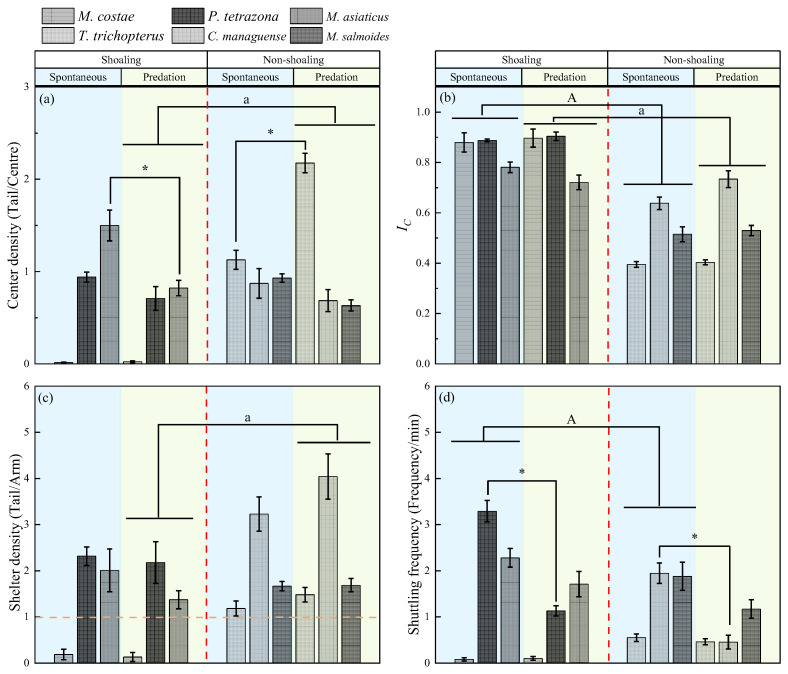
Changes in shoaling behavior parameters of fish with different shoaling preferences before and after simulated predation stimulation in the six-arm maze (mean ± S.E.). Notes: The capital letters (A) indicate significant differences between different shoaling-preference groups under spontaneous conditions (*p* < 0.05); The lowercase letter (a) indicate significant differences between different shoaling-preference groups after predation stimulation (*p* < 0.05). The star-shaped symbol (*) indicate significant differences within species before and after predation stimulation (*p* < 0.05).

**Center density**: The interaction between shoaling preference and predation stimulation had a significant effect on center density (*p* < 0.001). After predation stimulation, the center density of non-shoaling species was significantly higher than that of shoaling species (*p* < 0.05). Predation stimulation significantly increased center density in one non-shoaling species (*T. trichopterus*), but decreased center density in one shoaling species (*M. asiaticus*) and one non-shoaling species (*M. salmoides*) (*p* < 0.05) (Table 3, Figure 3b).

***I_C_***: Shoaling preference (*p* = 0.032) exerted a significant effect on *I_C_*, whereas neither predation stimulation nor its interaction with shoaling preference had a significant effect. Shoaling species exhibited significantly higher *I_C_* than non-shoaling species both before and after predation (*p* < 0.05) (Table 3, Figure 3b).

**Shelter density:** Neither shoaling preference, predation stimulation, nor their interaction had a significant effect on shelter density. After predation stimulation, non-shoaling species exhibited significantly higher shelter density than shoaling species (*p* < 0.05) (Table 3, Figure 3c).

**Shuttling frequency**: The effect of predation stimulation on shuttling frequency was significant (*p* < 0.001), whereas neither shoaling preference nor its interaction with predation stimulation showed any significant effects. Shoaling species exhibited significantly higher shuttling frequency than non-shoaling species both before and after predation. Predation stimulation significantly reduced shuttle frequency in one shoaling species (*P. tetrazona*) and two non-shoaling species (*C. managuense* and *M. salmoides*) (Table 3, Figure 3d).

## 4. Discussion

### 4.1. Anti-Predator Strategies in the Open-Water Conditions

In open-water environments, fish face higher predation pressure than in structured habitats, and shoaling behavior may become the optimal anti-predation strategy, depending on their shoaling preferences [32]. In our open-water arena, shoaling and non-shoaling species exhibited fundamentally different strategies under spontaneous conditions. Shoaling species maintained more cohesive and coordinated groups, characterized by significantly lower inter-individual distance, higher speed synchronization, and greater group polarity. They also exhibited elevated swimming activity (e.g., higher speed and acceleration) consistent with a strategy reliant on collective vigilance and coordinated escape in a structure-free habitat. In contrast, non-shoaling species showed significantly reduced locomotion (e.g., lower speed, acceleration, and percent time moving), suggesting a greater dependence on crypsis or individual vigilance rather than collective action.

After predation stimulation, two shoaling species (*M. costae* and *P. tetrazona*) increased their swimming activity, while the third (*M. asiaticus*) showed a slight decline—though its activity remained considerably higher than that of non-shoaling species. Shoal cohesion and coordination in shoaling species were largely maintained or only slightly diminished, with speed synchronization, polarity, and IID remaining significantly higher than in non-shoaling species. Interestingly, *M. costae* decreased its inter-individual distance despite the general tendency for spacing to increase during rapid escapes [33]. We suggest this may result from its enhanced acceleration, which facilitates fine-scale positional adjustments within the shoal, albeit at a higher energetic cost. Non-shoaling species, by contrast, displayed further reductions in swimming activity, group cohesion, and coordination after stimulation, building upon their already weaker baseline performance. Overall, shoaling species demonstrated superior swimming performance and group coordination across both spontaneous and threat conditions. Their ability to maintain tight group structure under predation risk reflects natural behavioral tendencies and likely represents a survival strategy that has evolved under long-term predation pressure. This aligns with established findings that shoaling improves predator avoidance, foraging efficiency, and information transfer [34,35,36]. For example, cohesive groups facilitate rapid threat detection and alarm propagation, leading to near-instantaneous collective escape [37]. Shoaling also reduces individual predation risk through dilution effects [6,38,39].

A comparison between two species with different shoaling preferences revealed that, under open-water conditions, both types showed reduced coordination during predation stimulation; however, their underlying strategies diverged fundamentally. Shoaling species prioritized rapid evasion by markedly increasing speed and acceleration, accepting a modest trade-off in group cohesion. This partial desynchronization during high-speed movement may enhance predator confusion and reduce targeting efficacy [35,40]. Non-shoaling species, constrained by limited locomotor capacity, could not execute such evasive maneuvers. Instead, they often remained motionless to avoid detection and conserve energy—a cryptic strategy that may allow them to await more favorable escape opportunities [41,42].

### 4.2. Anti-Predation Strategies in the Six-Arm Maze

When structural shelters are available, predation risk becomes spatially heterogeneous, providing fish with multiple anti-predator options. This habitat complexity offers a valuable context for assessing how social tendencies interact with environmental structure to shape anti-predator strategies. Shelters provide crucial protection against predators, serving an ecologically important function in natural environments [43,44]. Although most fish species tend to seek shelter when facing predation risk [45,46], the specific anti-predator strategies adopted can differ substantially among species. To systematically examine how fish with different levels of sociality respond to predation threats in structured habitats, this study employed a six-arm maze to compare collective anti-predator strategies between shoaling and non-shoaling species. Under spontaneous conditions, shoaling species exhibited high levels of collective activities and exploration, as indicated by high shuttle frequency and *I_C_* values. Notably, *M. costae* showed lower shoal shuttling frequency, possibly reflecting its inherently shy personality [47]. The lower shelter density and higher *I_C_* values observed in shoaling species suggest a consistent collective anti-predator strategy across different scenarios. In contrast, non-shoaling species demonstrated a stronger preference for shelter but exhibited lower group cohesion and shuttle frequency compared to shoaling species.

Following predation stimulation, shoaling species showed significant decreases in central density, shelter density, and shuttle frequency, while *I_C_* values remained stable. These findings indicate that shoaling species primarily rely on group cohesion as their core anti-predator strategy. However, non-shoaling species responded to predation stimulation by increasing shelter preference while reducing activity and exploration—a common strategy among solitary or benthic fish when confronting predators [48,49,50]. An exception was *T. trichopterus*, which displayed increased activity alongside decreased shelter preference and elevated central area density following stimulation. This response suggests that this species may rely on heightened local movement rather than shelter-seeking when threatened.

Overall, in complex environments, shoaling and non-shoaling species employ fundamentally different anti-predator strategies. Shoaling species maintain a defense strategy centered on coordinated group movement, exploiting the confusion effect to reduce individual predation risk through synchronized shoaling behavior [51]. In contrast, non-shoaling species primarily adopt a shelter-dependent strategy while minimizing movement to avoid detection. The interspecific variation observed among non-shoaling species (e.g., the distinct response of *T. trichopterus*) further highlights the context-dependent nature of anti-predator strategies. These behavioral differences are likely shaped by multiple factors, including body size, swimming performance, sensory capabilities, and ecological characteristics of their natural habitats (e.g., shelter availability, predator community composition). Collectively, these findings indicate that anti-predator strategies are not fixed traits but rather plastic responses shaped by long-term coevolution with predators and adaptation to specific ecological niches.

## 5. Conclusions

In conclusion, this study demonstrates that shoaling and non-shoaling fish species employ distinct anti-predator strategies. Shoaling species utilize “collective coordination,” maintaining group cohesion and executing rapid escapes to mitigate predation risk. In contrast, non-shoaling species depend on “shelter concealment” or reduced activity to minimize detection. These findings highlight that the interplay between habitat complexity and innate sociality jointly shapes the evolution of anti-predator behavior in fishes, providing a new theoretical framework for understanding how social behavior and environmental adaptation are linked in freshwater ecosystems.

## Figures and Tables

**Table 1:** Behavioral Parameters of Fish Swimming in Open Water.

Factor Being Measured	Formula	Variables Used in Formula
Instantaneous speed	$V\left(t\right)=\frac{\sqrt{{\left(x\left(t\right)-x\left(t-1\right)\right)}^{2}+{\left(y\left(t\right)-y\left(t-1\right)\right)}^{2}}}{d_{t}}$	where *x*(*t*) and *y*(*t*) are the *x*- and *y*-coordinates of each fish at time *t*, and *d_t_* is the time interval between consecutive frames (i.e., 1/15 s) [28].
Swimming acceleration	$a=v\left(t\right)-v(t-1)/d_{t}$	where *v*(*t*) and *v*(*t* − 1) are the velocities of each fish at times *t* and *t* − 1, respectively, and *d_t_* is the time interval between consecutive frames (i.e., 1/15 s).
Synchronization of speed	$S_{v}=1-\left|\frac{v_{i}-v_{j}}{v_{i}+v_{j}}\right|$	where *v_i_* and *v_j_* are the instantaneous speeds of the *i*-th and *j*-th fish in the group [29].
Inter-individual distance (IID)	$IID\left(t\right)=\frac{1}{n}\sum_{i\neq j}^{n}\sqrt{x_{i}\left(t\right)-x_{j}\left(t\right){)}^{2}+(y_{i}\left(t\right)-y_{j}(t){)}^{2}}$	where IID is the distance between individuals (cm), *x_i_* and *x_j_* are the abscissa values of the *i*-th and *j*-th fish in the shoal at time *t*, and *y_i_* and *y_j_* are the ordinate values of the *i*-th and *j*-th fish in the shoal at time *t*.
Polarity (*P*)	$P\left(t\right)=\frac{1}{n}\left|\sum_{i=1}^{n}v_{i}(t)\right|$	where *v_i_*(*t*) is the motion vector of the individual fish *i* per unit time, and the motion direction is from the coordinate point at time *t* − 1 to the position coordinate point at time *t*. *n* denotes the number of members of the population (i.e., *n* = 4 in this study).
Percent time spent moving (PTM)	$PTM=\frac{T_{1}}{T_{2}}\times 100$	When the swimming speed of fish exceeds 1.75 cm/s, it is regarded as moving state, and if the swimming speed is less than 1.75 cm/s, it is regarded as stationary state [30]. Where *T*_1_ represents the total time for fish to swim, and *T*_2_ represents the total video shooting time (spontaneous swimming time is 300 s, escape swimming time is 12 s).

**Table 2:** Results of mixed linear model (LMM) analysis of the effects of shoaling preference and simulated predation stimulation on shoaling behavior in open water (species as a random effect).

	Instantaneous Speed	Instantaneous Acceleration	Percent Time Spent Moving	Inter-Individual Distance	Synchronization of Speed	Polarity
Shoaling, S	*F*_1,4_ = 27.924 ***p* = 0.006** *	*F*_1,4_ = 23.536 ***p* = 0.008** *	*F*_1,4_ = 14.494 ***p* = 0.019** *	*F*_1,4_ = 7.890 ***p* = 0.048** *	*F*_1,4_ = 17.385 ***p* = 0.014** *	*F*_1,4_ = 7.496 *p* = 0.052
Predation, P	*F*_1,232_ = 1.243 *p* = 0.266	*F*_1,232_ = 6.161 ***p* = 0.014** *	*F*_1,232_ = 31.345 ***p* < 0.001** *	*F*_1,232_ = 6.212 ***p* = 0.013** *	*F*_1,232_ = 131.794 ***p* < 0.001** *	*F*_1,232_ = 86.613 ***p* < 0.001** *
S × P	*F*_1,232_ = 4.626 ***p* = 0.033** *	*F*_1,232_ = 13.32 ***p* < 0.001** *	*F*_1,232_ = 25.642 ***p* < 0.001** *	*F*_1,232_ = 4.313 ***p* = 0.039** *	*F*_1,232_ = 1.652 *p* = 0.200	*F*_1,232_ = 17.240 ***p* < 0.001** *

* Indicates a significant effect (*p* < 0.05).

**Table 3:** Results of mixed linear model analysis for the effects of shoaling preference and simulated predation stimulation on shoaling behavior in the six-arm maze (species as a random effect).

	Center Density	Shelter Density	*I_C_*	Shuttling Frequency
Shoaling, S	*F*_1,4.002_ = 0.820 *p* = 0.416	*F*_1,3.994_ = 0.794 *p* = 0.423	*F*_1,3.994_ = 10.503 ***p* = 0.032** *	*F*_1,4.004_ = 0.231 *p* = 0.656
Predation, P	*F*_1,228.002_ = 0.569 *p* = 0.452	*F*_1,227.994_ = 0.064 *p* = 0.801	*F*_1,227.995_ = 0.915 *p* = 0.340	*F*_1,227.004_ = 46.961 ***p* < 0.001** *
S × P	*F*_1,232_ = 13.666 ***p* < 0.001** *	*F*_1,232_ = 3.573 *p* = 0.060	*F*_1,227.995_ = 2.385 *p* = 0.124	*F*_1,228.004_ = 0.477 *p* = 0.491

* Indicates a significant effect (*p* < 0.05).

## Data Availability

The data presented in this study are available on request from the corresponding author. The data are not publicly available due to privacy/ethical restrictions.

## References

[1] Domenici P., Lefrançois C., Shingles A. (2007). Hypoxia and the Antipredator Behaviours of Fishes. Philos. Trans. R. Soc. B Biol. Sci..

[2] Kavaliers M., Choleris E. (2001). Antipredator Responses and Defensive Behavior: Ecological and Ethological Approaches for the Neurosciences. Neurosci. Biobehav. Rev..

[3] Ioannou C.C., Tosh C.R., Neville L., Krause J. (2008). The Confusion Effect—From Neural Networks to Reduced Predation Risk. Behav. Ecol..

[4] Polyakov A.Y., Quinn T.P., Myers K.W., Berdahl A.M. (2022). Group Size Affects Predation Risk and Foraging Success in Pacific Salmon at Sea. Sci. Adv..

[5] Doran C., Bierbach D., Lukas J., Klamser P., Landgraf T., Klenz H., Habedank M., Arias-Rodriguez L., Krause S., Romanczuk P. (2022). Fish Waves as Emergent Collective Antipredator Behavior. Curr. Biol..

[6] de Lima Mamede J., Nomura F. (2021). *Dendropsophus Minutus* (Hylidae) Tadpole Evaluation of Predation Risk by Fishing Spiders (*Thaumasia* Sp.: Pisauridae) Is Modulated by Size and Social Environment. J. Ethol..

[7] Sato H., Sakai Y., Kuwamura T. (2022). Effects of Group Behavior in the Predatory Raid on Damselfish Nests by the False Cleanerfish *Aspidontus taeniatus*. Ethology.

[8] Roberts G. (1996). Why Individual Vigilance Declines as Group Size Increases. Anim. Behav..

[9] Hammer T.L., Bize P., Gineste B., Robin J.-P., Groscolas R., Viblanc V.A. (2023). Disentangling the “Many-Eyes”, “Dilution Effect”, “Selfish Herd”, and “Distracted Prey” Hypotheses in Shaping Alert and Flight Initiation Distance in a Colonial Seabird. Behav. Process..

[10] Zhao M., Feng G., Wang H., Shen C., Fu Y., Zhang Y., Zhang H., Yao Y., Chen J., Xu W. (2024). The Influence of Shelter Type and Coverage on Crayfish (*Procambarus clarkii*) Predation by Catfish (*Silurus asotus*): A Controlled Environment Study. Animals.

[11] Ren Y., Xie D., Li B., Liu Y., Hu S., Liu H., Shi Y., Zhu S. (2020). Influence of Water Temperature, Habitat Complexity and Light on the Predatory Performance of the Dark Sleeper *Odontobutis potamophila* (Günther, 1861). J. Freshwater Ecol..

[12] Orpwood J.E., Magurran A.E., Armstrong J.D., Griffiths S.W. (2008). Minnows and the Selfish Herd: Effects of Predation Risk on Shoaling Behaviour Are Dependent on Habitat Complexity. Anim. Behav..

[13] Rilov G., Figueira W.F., Lyman S.J., Crowder L.B. (2007). Complex Habitats May Not Always Benefit Prey: Linking Visual Field with Reef Fish Behavior and Distribution. Mar. Ecol. Prog. Ser..

[14] Fakan E.P., Allan B.J.M., Illing B., Hoey A.S., McCormick M.I. (2023). Habitat Complexity and Predator Odours Impact on the Stress Response and Antipredation Behaviour in Coral Reef Fish. PLoS ONE.

[15] Lennox R.J., Kambestad M., Berhe S., Birnie-Gauvin K., Cooke S.J., Dahlmo L.S., Eldøy S.H., Davidsen J.G., Hanssen E.M., Sortland L.K. (2025). The Role of Habitat in Predator–Prey Dynamics with Applications to Restoration. Restor. Ecol..

[16] Mukherjee I., Bhat A. (2024). The Impact of Predators and Vegetation on Shoaling in Wild Zebrafish. R. Soc. Open Sci..

[17] Encel S.A., Schaerf T.M., Lizier J.T., Ward A.J. (2021). Locomotion, Interactions and Information Transfer Vary According to Context in a Cryptic Fish Species. Behav. Ecol. Sociobiol..

[18] Liu L., Zhang R., Wang X., Zhu H., Tian Z. (2020). Transcriptome Analysis Reveals Molecular Mechanisms Responsive to Acute Cold Stress in the Tropical Stenothermal Fish Tiger Barb (*Puntius tetrazona*). BMC Genom..

[19] Ramos T.P.A., Lustosa-Costa S.Y., Lima R.M.O., Barbosa J.E.D.L., Menezes R.F. (2021). First Record of *Moenkhausia Costae* (Steindachner 1907) in the Paraíba Do Norte Basin after the São Francisco River Diversion. Biota Neotrop..

[20] Wu J., Chen Y., Li C., Song Z. (2022). Hatchery Release Programme Modified the Genetic Diversity and Population Structure of Wild Chinese Sucker (*Myxocyprinus asiaticus*) in the Upper Yangtze River. Aquat. Conserv. Mar. Freshwater Ecosyst..

[21] Yu H., Lin Z., Xiao F. (2024). Role of Body Size and Shape in Animal Camouflage. Ecol. Evol..

[22] Degani G. (2020). Brain Control Reproduction by the Endocrine System of Female Blue Gourami (*Trichogaster trichopterus*). Biology.

[23] Ai C., Chen X., Zhong Z., Jiang Y. (2020). Physiological Responses to Salinity Stress in the Managua Cichlid, *Cichlasoma managuense*. Aquacult. Res..

[24] (2001). Laboratory Animal—Requirements of Environment and Housing Facilities.

[25] Pérez-Escudero A., Vicente-Page J., Hinz R.C., Arganda S., De Polavieja G.G. (2014). idTracker: Tracking Individuals in a Group by Automatic Identification of Unmarked Animals. Nat. Methods.

[26] Romero-Ferrero F., Bergomi M.G., Hinz R.C., Heras F.J., De Polavieja G.G. (2019). Idtracker. Ai: Tracking All Individuals in Small or Large Collectives of Unmarked Animals. Nat. Methods.

[27] Jolles J.W., Boogert N.J., Sridhar V.H., Couzin I.D., Manica A. (2017). Consistent Individual Differences Drive Collective Behavior and Group Functioning of Schooling Fish. Curr. Biol..

[28] Fu S. (2016). Effects of Group Size on Schooling Behavior in Two Cyprinid Fish Species. Aquat. Biol..

[29] Delcourt J., Poncin P. (2012). Shoals and Schools: Back to the Heuristic Definitions and Quantitative References. Rev. Fish Biol. Fish..

[30] Tang Z., Huang Q., Wu H., Kuang L., Fu S.-J. (2017). The Behavioral Response of Prey Fish to Predators: The Role of Predator Size. PeerJ.

[31] Li W., Li J., Fu S. (2024). Effects of Starve and Shelter Availability on the Group Behavior of Two Freshwater Fish Species (*Chindongo demasoni* and *Spinibarbus sinensis*). Animals.

[32] Ioannou C.C., Laskowski K.L. (2023). A Multi-Scale Review of the Dynamics of Collective Behaviour: From Rapid Responses to Ontogeny and Evolution. Phil. Trans. R. Soc. B.

[33] Mizumoto N., Miyata S., Pratt S.C. (2019). Inferring Collective Behaviour from a Fossilized Fish Shoal. Proc. R. Soc. B.

[34] Bai Y., Tang Z.-H., Fu S.-J. (2019). Numerical Ability in Fish Species: Preference between Shoals of Different Sizes Varies among Singletons, Conspecific Dyads and Heterospecific Dyads. Anim. Cogn..

[35] Nadler L.E., McCormick M.I., Johansen J.L., Domenici P. (2021). Social Familiarity Improves Fast-Start Escape Performance in Schooling Fish. Commun. Biol..

[36] Demandt N., Bierbach D., Kurvers R.H.J.M., Krause J., Kurtz J., Scharsack J.P. (2021). Parasite Infection Impairs the Shoaling Behaviour of Uninfected Shoal Members under Predator Attack. Behav. Ecol. Sociobiol..

[37] Zhao Z., Shi Q., Liu Y. (2025). Modeling and Simulation of the Fish Collective Behavior with Risk Perception and Startle Cascades. Phys. A.

[38] Horka P., Vlachova M. (2023). The Effect of Turbidity on the Behavior of Bleak (*Alburnus alburnus*). Fishes.

[39] Aguiñaga J., Jin S., Pesati I., Laskowski K.L. (2024). Behavioral Responses of a Clonal Fish to Perceived Predation Risk. PeerJ.

[40] Larsson M. (2024). Schooling Fish from a New, Multimodal Sensory Perspective. Animals.

[41] Penry-Williams I.L., Brown C., Ioannou C.C. (2025). Visual Lateralisation in Female Guppies *Poecilia reticulata* Demonstrates Social Conformity but Is Reduced When Observing a Live Predator *Andinoacara pulcher*. Ecol. Evol..

[42] Loftus S., Borcherding J. (2017). Does Social Context Affect Boldness in Juveniles?. Curr. Zool..

[43] Li X., Zhu Y., Chen S., Zhu T., Wu X., Li X. (2025). Behavioral Characteristics of Largefin Longbarbel Catfish *Hemibagrus macropterus*: Effects of Sex and Body Size on Aggression and Shelter Selection. Animals.

[44] Gesto M., Jokumsen A. (2022). Effects of Simple Shelters on Growth Performance and Welfare of Rainbow Trout Juveniles. Aquaculture.

[45] Zhang H., Zhu B., Yu L., Liu D., Wang F., Lu Y. (2021). Selection of Shelter Shape by Swimming Crab (*Portunus trituberculatus*). Aquacult. Rep..

[46] Zhang H., Zhu B., Yu L., Wang F. (2022). Shelter Color Selection of Juvenile Swimming Crabs (*Portunus trituberculatus*). Fishes.

[47] Gartland L.A., Firth J.A., Laskowski K.L., Jeanson R., Ioannou C.C. (2022). Sociability as a Personality Trait in Animals: Methods, Causes and Consequences. Biol. Rev..

[48] Zhang Y., Zhou W., Fu S., Nadler L.E., Killen S.S., Zhou K.-Y., Zheng S.-L., Fu C. (2025). Social Conditions Alter the Growth, Metabolic Rate, Behavioral Traits, and Food-Shelter Preferences in the Juvenile Qingbo *Spinibarbus sinensis*. Behav. Ecol. Sociobiol..

[49] Huijbers C., Nagelkerken I., Govers L., van de Kerk M., Oldenburger J., de Brouwer J. (2011). Habitat Type and Schooling Interactively Determine Refuge-Seeking Behavior in a Coral Reef Fish throughout Ontogeny. Mar. Ecol. Prog. Ser..

[50] Slavík O., Maciak M., Horký P. (2012). Shelter Use of Familiar and Unfamiliar Groups of Juvenile European Catfish *Silurus glanis*. Appl. Anim. Behav. Sci..

[51] Ioannou C.C., Guttal V., Couzin I.D. (2012). Predatory Fish Select for Coordinated Collective Motion in Virtual Prey. Science.

